# Effects of Fishing and Fishing Closures on Beach Clams: Experimental Evaluation across Commercially Fished and Non-Fished Beaches before and during Harvesting

**DOI:** 10.1371/journal.pone.0146122

**Published:** 2016-01-05

**Authors:** Charles A. Gray

**Affiliations:** WildFish Research, Grays Point, Sydney, Australia; University of Minnesota, UNITED STATES

## Abstract

Management responses to reconcile declining fisheries typically include closed areas and times to fishing. This study evaluated this strategy for a beach clam fishery by testing the hypothesis that changes in the densities and size compositions of clams from before to during harvesting would differ between commercially fished and non-fished beaches. Sampling was spatially stratified across the swash and dry sand habitats on each of two commercially fished and two non-fished beaches, and temporally stratified across three six-week blocks: before, early and late harvesting. Small-scale spatio-temporal variability in the densities and sizes of clams was prevalent across both habitats and the components of variation were generally greatest at the lowest levels examined. Despite this, differences in the densities and sizes of clams among individual beaches were evident, but there were few significant differences across the commercially fished versus non-fished beaches from before to during harvesting. There was no evidence of reduced densities or truncated size compositions of clams on fished compared to non-fished beaches, contrasting reports of some other organisms in protected areas. This was probably due to a combination of factors, including the current levels of commercial harvests, the movements and other local-scale responses of clams to ecological processes acting independently across individual beaches. The results identify the difficulties in detecting fishing-related impacts against inherent levels of variability in clam populations. Nevertheless, continued experimental studies that test alternate management arrangements may help refine and determine the most suitable strategies for the sustainable harvesting of beach clams, ultimately enhancing the management of sandy beaches.

## Introduction

Fishing has had detrimental impacts on wild populations and assemblages of aquatic organisms of various phyla across a spectrum of habitats throughout the world [1–4]. In particular, many harvested species have experienced substantial population declines as well as changes in demographic characteristics such as truncation of size and age composition, reduced sizes and ages at reproduction, altered growth rates and mortality schedules [5–10]. The impacts and responses of organisms to fishing can vary considerably depending on the type, intensity and history of fishing activities, as well as the life history characteristics and resilience of individual species and populations [9,11,12].

Initiatives to reconcile the effects of fishing and provide greater protection to wild organisms and habitats include areas and times either fully or partially closed to fishing [13,14], fishing gear restrictions and modifications [15], catch and bycatch quotas and size and bag limits [16]. Several such measures have been shown to be effective across different fisheries and landscapes. For example, no-take fishing areas can restore densities and size compositions of harvested species, and help maintain ecosystem biodiversity and functioning [14,17–19]. Similarly, modifications to fishing gears can reduce levels of catches of unwanted species as well as damage to habitats [20]. Nevertheless, in many cases the effects of implemented management arrangements have not been tested. Ideally, the success or failure of such management measures should be evaluated experimentally as part of an adaptive management regime [21,22].

Beach clams (Bivalvia: Donacidae, Mesodermatidae, Veneridae) are harvested for food and bait on sandy beaches worldwide [23,24], but because they primarily inhabit the intertidal and shallow subtidal they are easily accessible and relatively simple and cheap to harvest, making them readily susceptible to over exploitation [24]. Indeed, populations of several species have over relatively short periods of time been depleted [23,24], a trend observed for many other exploited invertebrates [25]. This scenario could also be true for the Australian beach clam *Donax deltoides* [26,27]. For example, in the state of New South Wales (NSW) alone, following the developmental phase of the fishery in the 1950s total reported commercial landings of *D*. *deltoides* increased to peak at 670,000 kilograms (kg) in 2001, after which it fell (along with commercial catch-per-unit-effort) to only 9,000 kg in 2011, despite increasing product prices and markets [28]. Throughout this time, recreational and indigenous catches were unrestricted and unchecked but were probably large across many beaches [29,30]. Although the reasons for the rapid decline in commercial catches and catch rates are unclear, fishing was probably a contributing factor [26].

Management responses to declining beach clam fisheries have usually included closed areas and times to fishing, to varying degrees of success (i.e. when actually tested) [23,24,31]. In response to the fall in commercial catches and other broader population declines of *D*. *deltoides*, several management initiatives designed to reduce fishing effort and harvest and stabilize the fishery, and therefore halt further population declines, were introduced to the NSW fishery in 2012. The strategy incorporated a six-month total commercial fishing closure, spatially explicit commercial fishing closures of whole beaches and specific zones along particular beaches, a maximum daily catch quota of 40 kg per-commercial fisher, as well as the introduction of a minimum legal size limit (45 mm shell length, SL). Recreational and indigenous fishers remain permitted to catch clams year round across all beaches, but due to concerns over toxins they can now only use clams as bait in-situ and cannot remove them from beaches. Because of this, the combined harvest from these two sectors is estimated to be small and may be as little as 5% the commercial harvest [28,29]. The harvesting of clams by all sectors is restricted to digging by hand, with no mechanical apparatus permitted.

This study was done in response to the above management arrangements being implemented in the NSW commercial beach clam fishery and the first to examine the potential impacts of fishing on beach clams by comparing populations across beaches open and closed to commercial fishing, both before and during the harvesting season. The specific hypothesis tested was that changes in the densities and size compositions of *D*. *deltoides* from before to during (early and late) the six month harvesting season would differ between commercially fished and non-fished beaches. It was predicted that the densities of clams would decline and their size compositions become truncated throughout the fishing season on commercially fished compared to non-fished beaches.

## Materials and Methods

### Experimental design and sampling

No specific permissions or permits were required to access and sample the study beaches as they had full public access. The field sampling did not involve endangered or protected species.

This study was done in 2013 across four high-energy ocean sandy beaches in eastern Australia: Ten Mile (latitude, longitude: -29.29, 153.35; length: 28.5 km), Sandon (-29.64, 153.32; 7.3 km), Illaroo (-29.72, 153.30; 9.2 km) and Smoky (-30.98, 153.05; 16.0 km). Each beach is enclosed between rocky headlands, fronted by rip-dominated bar systems and exposed to seas from the north, east and south directions [32]. Sandon and Illaroo have a history of sporadic commercial harvesting of clams and were closed to harvesting throughout the study. In contrast, both Ten Mile and Smoky have a strong history of continuous commercial harvesting for clams and were open to commercial clam harvesting between 1 June and 30 November 2013.

Before sampling started, commercial fishers identified Ten Mile and Smoky among others as key clam harvesting beaches for 2013. Choice of non-fished beaches was from a greater source and based on geographic location, suitable length, accessibility and presence of clams. Scoping of numerous beaches in March/April 2013 identified that several non-fished beaches had no discernable populations of clams, whereas they were relatively abundant across all study beaches prior to sampling.

Sampling of clams on each beach was stratified temporally across three discrete periods, before and during the six-month commercial harvesting season in 2013. The length of each sampling period and the interval between consecutive sampling periods was 6 weeks. Period 1 (before) was in April/ May when all beaches were totally closed to commercial clam harvesting. Period 2 (early harvesting) was in July/August and Period 3 (late harvesting) was in October/ November with sampling beginning 6 and 18 weeks, respectively, after the commencement of harvesting on 1 June 2013.

Clams were sampled across two habitats, the swash zone and the dry sand belt typically located between 10 and 30 m above the low-tide swash zone level on each beach. To account for small-scale temporal and spatial variability [33], in each of the three periods sampling was done across two randomly selected days in each of three randomly selected weeks, except for the swash habitat in Period 1 when only four days (two weeks) were sampled. On each sampling day, eight sites in the swash zone and another eight sights in the identified clam belt in the dry sand were selected at random on each beach, and at each of these locations, six replicate samples were taken [27]. A total of 96 samples were therefore collected each day of sampling on each beach. Sampling was done during daytime within 3 hours either side of low tide [27] and it took approximately 4 hours to complete sampling each day.

Different sampling methods were used to sample clams in each habitat. Clams in the swash zone were sampled by finger digging for 30 sec a small area (average diameter 57 cm, depth 18 cm) of sand and scooping it into a net that had 12 mm mesh hung on a frame measuring 35 x 21cm [27]. Clams in the dry sand habitat were sampled by excavating sand to a depth of 20 cm within a square box quadrat that had 32 cm sides [34], after which the excavated sand was sieved through a bag with 6 mm mesh. Density of clams was therefore expressed as number sampled per 30 sec dig in the swash and per quadrat in the dry. All clams collected in each replicate sample were counted and measured for shell length (SL, mm) and operational information including time of sampling and beach and sea conditions were recorded. The same technician team sampled across all beaches.

### Data Analyses

Permutation-based analyses of variance (PERMANOVA) [35] were used to test whether the densities and sizes of clams differed between commercially fished and non-fished beaches from before to during the harvesting season. PERMANOVA is appropriate to analyse univariate and multivariate data in response to factors or treatments in an experimental design. Because it is permutation-based, it is generally free of assumptions concerning data normality that often plague standard parametric statistics and was therefore an appropriate and robust analytical technique for the study data [35,36].

Separate univariate analyses were performed for densities of total, legal and sublegal sized clams and each analysis was based on the Euclidean distance matrix with Type III (partial) sums of squares. Use of the Euclidean distance matrix meant that construction of F-ratios was equivalent to traditional statistics [37]. However, the actual P-values were obtained by performing 999 unrestricted permutations of the raw data for each term in each analysis (P(perm)) and also using Monte Carlo simulations (P(MC)) [37]. The P(MC) values are most appropriate when the total number of possible permutations and unique outcomes is low [37]. This applied to the test for the factor ‘Beach Management Type’ in the analyses reported here (see below).

For the density data, the specific five-factor model used in each PERMANOVA was: Beach Management Type (i.e. commercially fished v non-fished—fixed), Beach (nested in Beach Management Type—random), Period (Before, Early & Late harvest—fixed), Day (nested in Period and Beach—random), Site (nested in Beach, Day and Period–random). Separate analyses were done for each habitat (swash and dry sand) because they were sampled in different ways. In these analyses, significant Beach Management Type x Period (degrees of freedom = 2,4; P-value permutations = 999), and Beach (BMT) x Period (degrees of freedom = 4,52 or 4,60; P-value permutations = 999) interactions potentially identified fishing-related effects on clams. The proportion of variation attributable to each term in each PERMANOVA model was calculated to aid interpretation of results [36,37]. All negative variation component values were treated as zero, eliminated from the analysis and the remaining variation components recalculated [38]. Each component directly estimated variability for each term independent of the other terms. All analyses were done using the PRIMER 6—PERMANOVA^+^ program [37].

Fishing and other anthropogenic perturbations can not only impact actual densities of organisms, but also potentially affect levels of variability in densities [39,40]. This was investigated here by examining whether the components of variation of total clam densities differed across the commercially fished versus the non-fished beaches from before to during harvesting. For each habitat and beach, the components of variation attributed to the factor Site were determined separately for each sampling day using a one factor PERMANOVA that compared densities across the eight sampled sites. Each of these values was converted to a proportion for standardization and then used as a replicate (i.e. day) value for each respective period in a 3-factor PERMANOVA that had the factors Beach Management Type (commercially fished v non-fished—fixed), Beach (nested in Beach Management Type–random) and Period (fixed). A separate analysis was done for each habitat. As detailed above, each analysis was based on the Euclidean distance matrix and Type III (partial) sums of squares, with 999 unrestricted permutations of the raw data and Monte Carlo simulations used to calculate the P-values [37].

PERMANOVA was also used to test whether the size compositions of sampled populations of clams differed between commercially fished and non-fished beaches across sampling periods. To reduce the number of size classes that contained zero clams, the proportion of clams in each 5 mm size class was used to classify samples. Further, because *D*. *deltoides* were not always sampled in large densities at each site on each sampling day, data were pooled across all eight sites sampled on each day on each beach. Days when no clams were caught were omitted from the analyses. The total size composition data for each day were then used as replicates for each period so that the analytical design for each analysis was: Beach Management Type (commercially fished v non-fished—fixed), Beach (nested in Beach Management Type–random) and Period (fixed). Separate analyses were done for the swash and dry habitats on each beach and each analysis was based on the Euclidean distance matrix with Type III (partial) sums of squares. Separate P-values were determined by performing 999 unrestricted permutations of the raw data and Monte Carlo simulations [37]. As above, the same two interaction terms were most important in identifying potential effects of fishing

## Results

### Densities of clams

A total of 6,056 and 5,610 clams were sampled across the commercially fished and non-fished beaches, respectively (Table 1). Greater numbers of total clams were sampled in the swash compared to the dry habitat across each beach.

**Table 1:** The total numbers of clams sampled in the swash and dry habitats across the two commercially fished and non-fished beaches throughout the study.

Beach	Dry habitat	Swash habitat
Commercially fished		
Ten Mile	916	1650
Smoky	1310	2180
Non-fished		
Sandon	542	921
Illaroo	1134	3013

**Fig 1:**
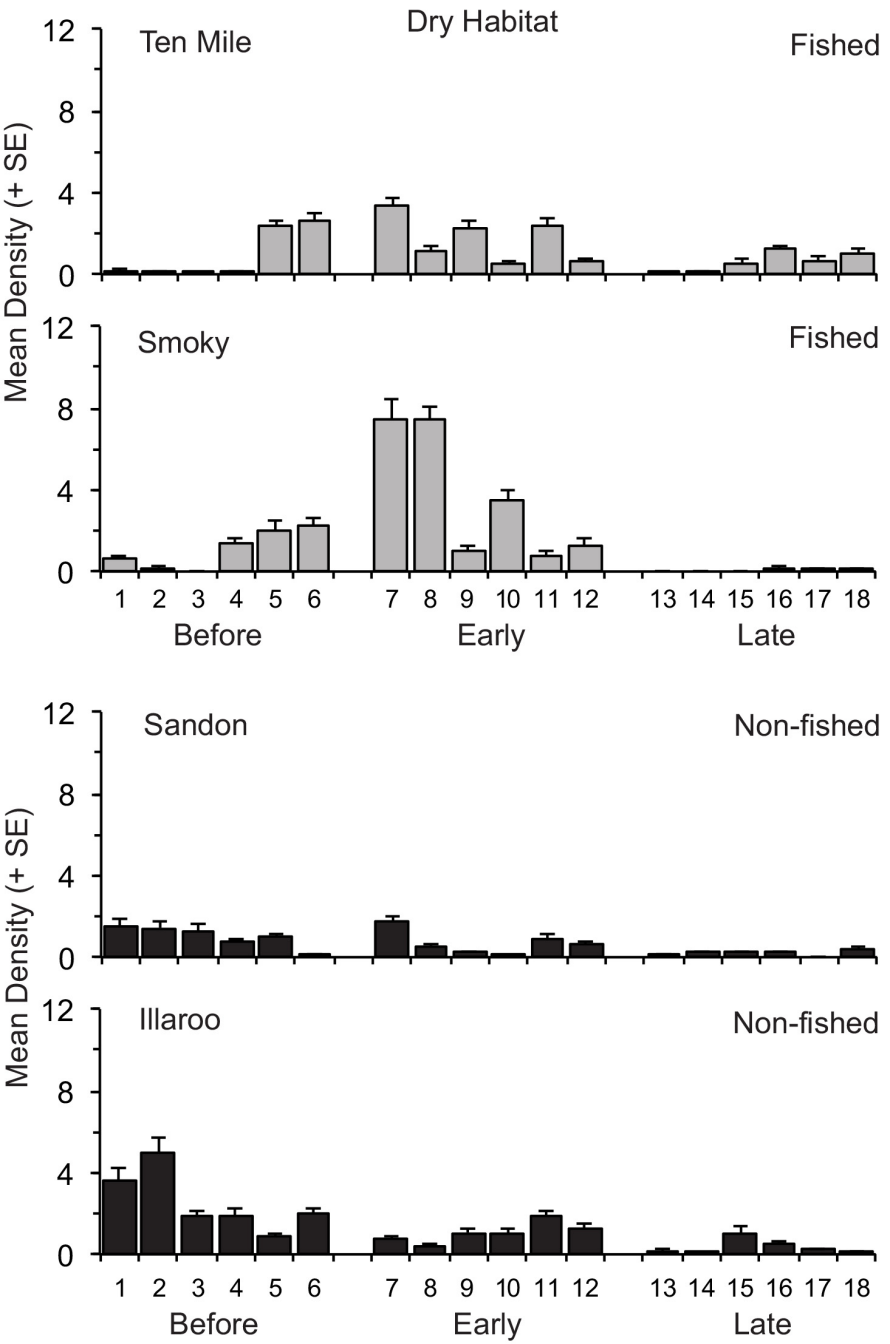
Mean (+ SE) density of *Donax deltoides* sampled in the dry habitat on each of six days before, early and late harvesting across the two commercially fished and non-fished beaches

The significance of the permutation-based and Monte Carlo derived P-values was mostly similar for each term in each model (Table 2). There was only one significant Beach Management Type x Period interaction and that was for densities of legal clams in the dry habitat (Table 2). The pairwise tests could not distinguish significant differences among groups due to the low number (3) of available permutations. Nevertheless, the data in Fig 1 indicated that densities of clams were greater across the two fished beaches compared to the non-fished beaches early harvesting, whereas no such patterns were apparent before or late harvesting. Beach Management Type and its interaction with Period explained less than 3% of total variation in each analysis, except for total and legal clams in the dry (Table 2).

**Table 2:** Results of univariate PERMANOVAs comparing the densities of total, legal and sublegal clams across commercially fished and non-fished beaches before, early and late harvesting.

A. Dry Habitat		Total clams						Legal clams						Sublegal clams					
Source	df	MS	Pseudo-F	P(perm)	Unique Perms	P(MC)	CV%	MS	Pseudo-F	P(perm)	Unique Perms	P(MC)	CV%	MS	Pseudo-F	P(perm)	Unique Perms	P(MC)	CV%
Beach Management Type	1, 2	87.529	0.598	0.646	3	0.501	0.0	102.090	6.571	0.336	3	0.123	1.9	0.560	0.006	1.000	3	0.946	0.0
Period	2, 4	644.650	4.669	0.106	999	0.092	7.1	289.060	9.512	**0.010**	999	**0.033**	8.4	77.063	1.449	0.300	998	0.304	1.3
Beach(BMT)	2, 60	146.330	2.052	0.170	999	0.138	1.4	15.538	0.507	0.636	998	0.584	0.0	89.433	8.104	**0.001**	999	**0.002**	5.8
BMT x Period	2, 4	464.450	3.364	0.117	999	0.141	9.1	222.690	7.328	**0.021**	999	0.057	12.5	61.783	1.162	0.385	999	0.417	1.0
Beach(BMT) x Period	4, 60	138.080	1.937	0.112	999	0.107	3.7	30.389	0.992	0.447	998	0.444	0.0	53.190	4.820	**0.001**	999	**0.002**	9.4
Day(Beach(BMT) x Period)	60, 504	71.298	6.624	**0.001**	998	**0.001**	20.2	30.633	6.931	**0.001**	997	**0.001**	20.4	11.036	3.915	**0.001**	996	**0.001**	11.0
Site(Day(Beach(BMT) x Period))	504, 2880	10.764	4.838	**0.001**	998	**0.001**	22.8	4.420	4.722	**0.001**	995	**0.001**	21.7	2.819	3.658	**0.001**	996	**0.001**	21.9
Residual	2880	2.225					35.7	0.936					35.0	0.771					49.5
Total	3455																		
B. Swash Habitat		Total clams						Legal clams						Sublegal clams					
Source	df	MS	Pseudo-F	P(perm)	Unique Perms	P(MC)	CoV%	MS	Pseudo-F	P(perm)	Unique Perms	P(MC)	CoV%	MS	Pseudo-F	P(perm)	Unique Perms	P(MC)	CoV%
Beach Management Type	1, 2	16.254	0.009	0.667	3	0.94	0.0	362.520	3.523	0.345	3	0.214	2.6	532.300	0.530	0.649	3	0.554	0.0
Period	2, 4	1597.100	4.484	0.096	999	0.092	5.9	440.950	3.892	0.127	998	0.113	4.8	468.030	3.755	0.122	998	0.133	3.9
Beach(BMT)	2, 52	1737.100	12.708	**0.001**	998	**0.001**	10.4	102.890	1.690	0.200	998	0.193	0.8	1003.600	32.049	**0.001**	998	**0.001**	14.9
BMT x Period	2, 4	108.340	0.304	0.778	999	0.738	0.0	14.477	0.128	0.858	999	0.891	0.0	132.970	1.067	0.418	999	0.409	0.2
Beach(BMT) x Period	4, 52	356.210	2.606	**0.045**	999	**0.036**	4.2	113.310	1.861	0.139	999	0.124	3.1	124.640	3.981	**0.006**	999	**0.012**	4.2
Day(Beach(BMT) x Period)	52, 448	136.700	3.133	**0.001**	997	**0.001**	9.3	60.891	4.482	**0.001**	995	**0.001**	14.6	31.313	1.498	**0.018**	998	**0.016**	2.5
Site(Day(Beach(BMT) x Period))	448, 2560	43.632	4.925	**0.001**	996	**0.001**	27.8	13.584	4.164	**0.001**	996	**0.001**	25.6	20.903	5.624	**0.001**	998	**0.001**	32.4
Residual	2560	8.860					42.5	3.262					48.4	3.717					42.1
Total	3071																		

df = degrees of freedom, MS = mean square, Pseudo-F = F-ratio, P(perm) = permutation based P-value, Unique perms = number of unique permutations, P(MC) = Monte Carlo simulation P-value, CV% = component of variation percentage

The Beach Management Type x Period and Beach(BMT) x Period interaction terms if significant identify possible effects of fishing and management zoning.

**Fig 2:**
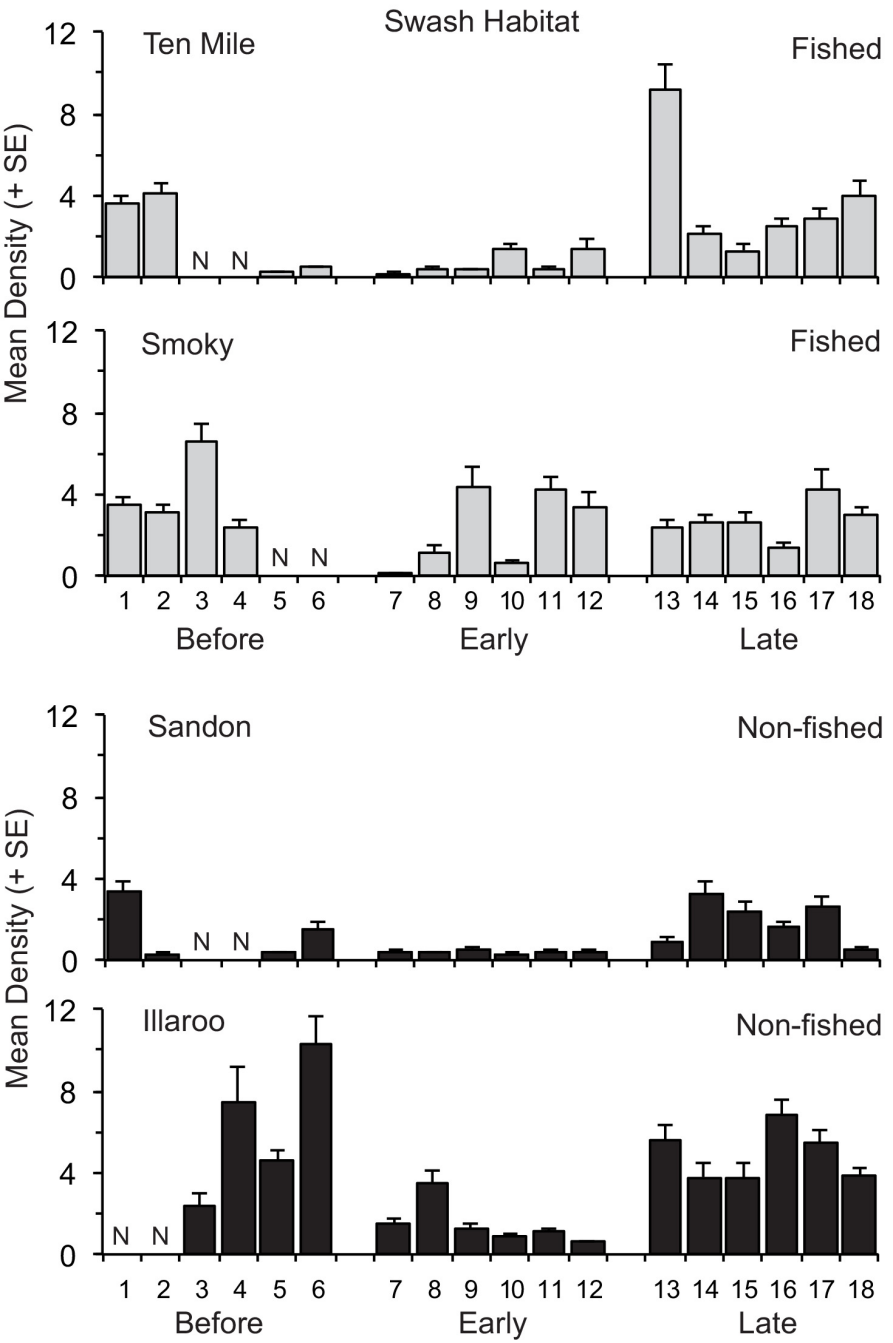
Mean (+ SE) density of *Donax deltoides* sampled in the swash habitat on each of four days before, and six days early and late harvesting across the two commercially fished and non-fished beaches. N denotes not sampled.

The densities of total and sublegal clams in the swash and sublegal clams in the dry significantly differed according to the Beach(BMT) x Period interaction (Table 2). These significant interactions indicated that changes in densities between periods were not always the same for both fished or non-fished beaches. For example, the pairwise tests identified that for the commercially fished beaches, the densities of total clams in the swash on Smoky did not significantly differ between periods, whereas on Ten Mile total clam densities during late harvest were significantly greater than early harvest, but not before harvest (Fig 2). For the non-fished beaches, total densities in the swash were significantly lower early harvest than before or late harvest on Illaroo, but only less than late harvest on Sandon (Fig 2). Similarly, the densities of sublegal clams in the dry did not significantly differ between periods on Ten Mile, but were significantly greater late harvest than either before or early harvest on Smoky.

Densities of total, sublegal and legal sized clams consistently differed significantly according to the factors Site and Day (Table 2). These results demonstrate there was significant variability in densities from site-to-site on each beach on each sampling day, as well as among individual sampling days within each period on each beach (Figs 1 and 2). Across both habitats there was also considerable small-scale variability in densities of clams among replicate samples taken at each site on each sampling day: in each analysis the components of variation were consistently greatest for the residual, accounting for 35 to 49% of total variation (Table 2).

Across both the swash and dry habitats the components of variation attributed to the factor Site did not significantly differ according to either the Beach Management Type x Period interaction (PERMANOVA, Swash: d.f. = 2,4, MS = 0.034, P(perm) = 0.677; Dry: d.f. = 2,4, MS = 0.035, P(perm) = 0.645) or the Beach(BMT) x Period interaction (PERMANOVA, Swash: d.f. = 4,52, MS = 0.076, P(perm) = 0.068; Dry: d.f. = 4,60, MS = 0.074, P(perm) = 0.212). Thus, there were no detectable effects of harvesting on levels of variability of total clams.

### Sizes of clams

**Fig 3:**
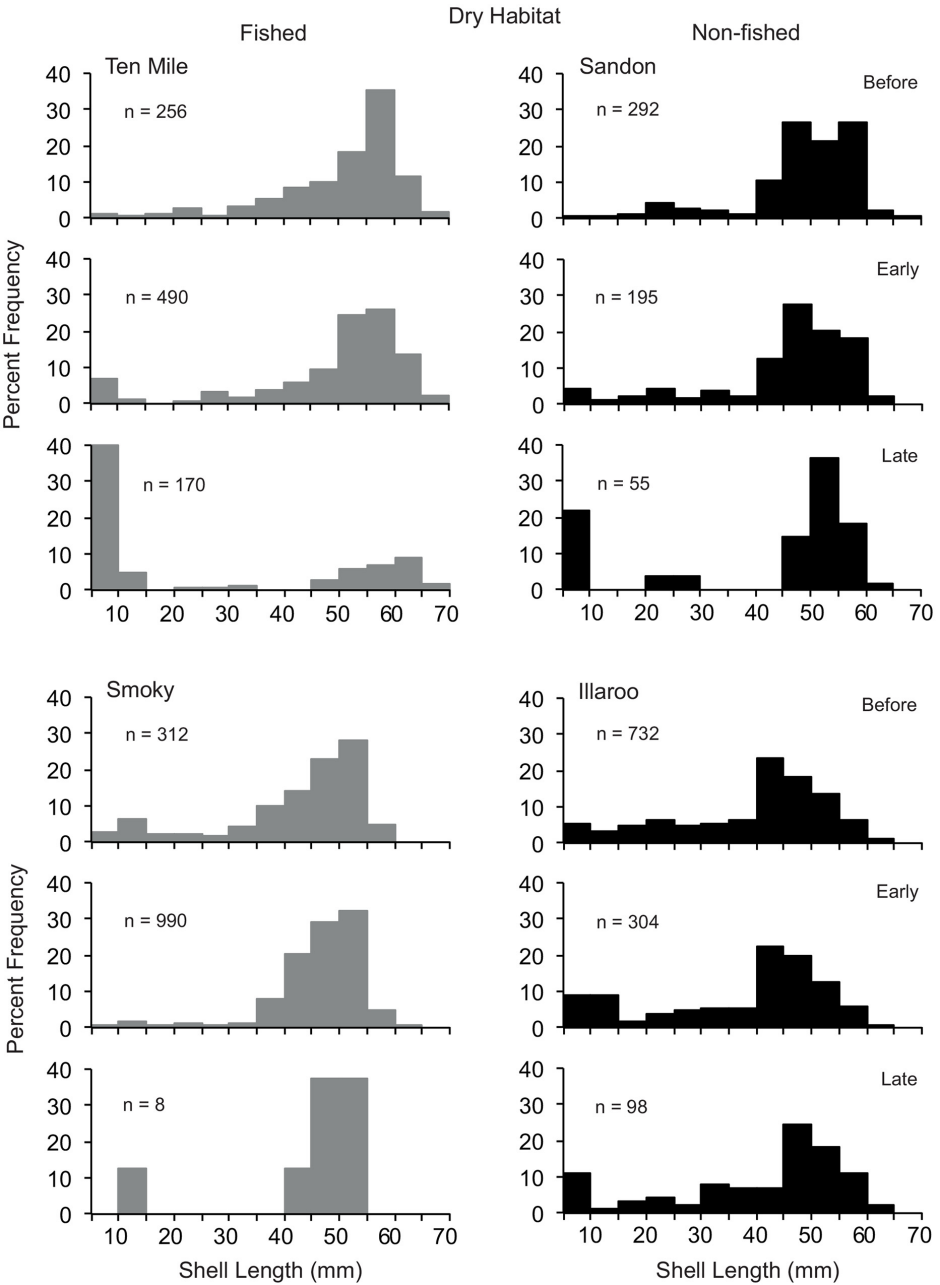
Size compositions of *Donax deltoides* sampled in the dry habitat before, early and late harvesting across the two commercially fished and non-fished beaches

**Fig 4:**
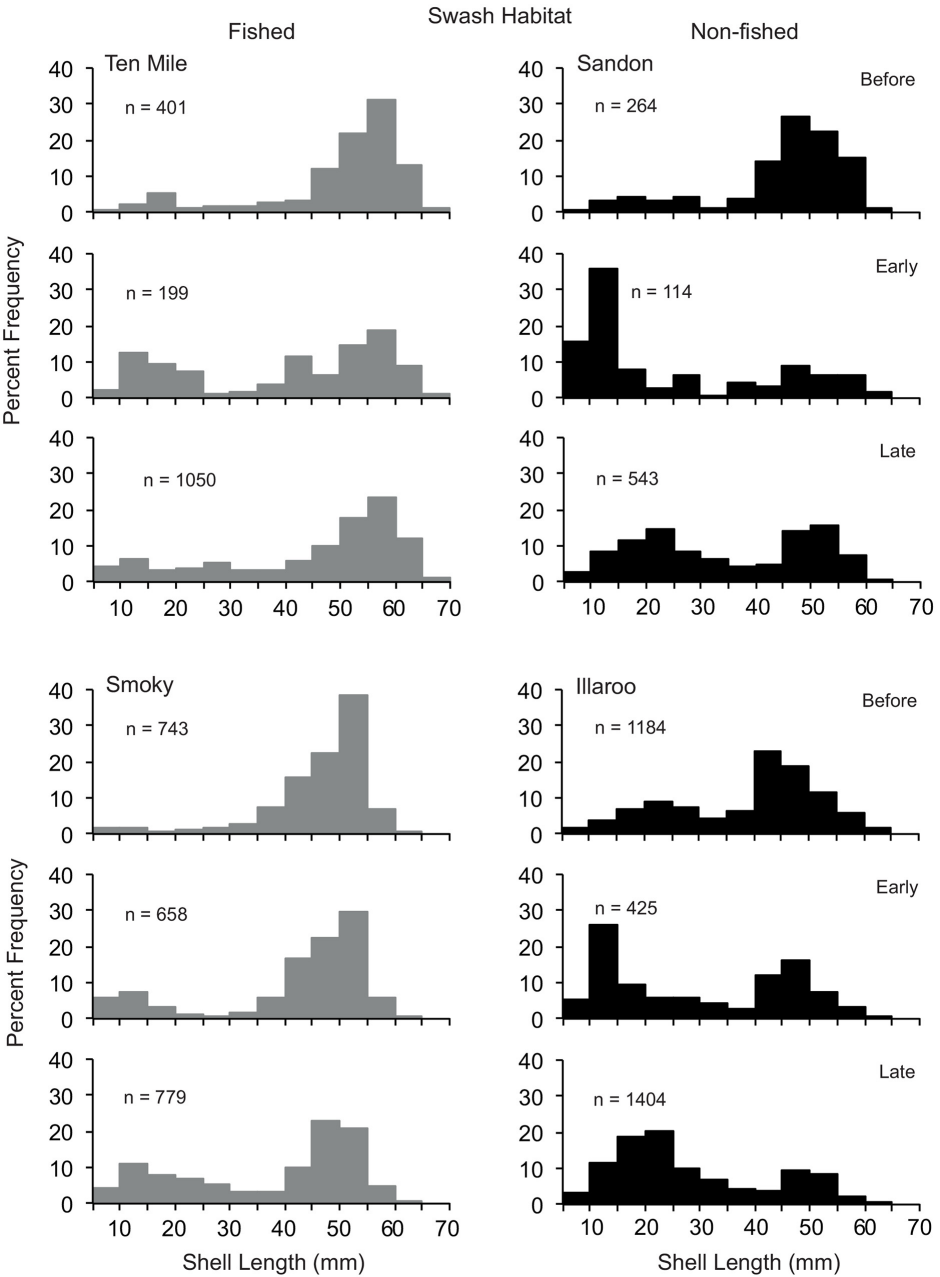
Size compositions of *Donax deltoides* sampled in the swash habitat before, early and late harvesting across the two commercially fished and non-fished beaches

PERMANOVA identified that across both habitats there were significant differences in the size compositions of clams according to the Beach(BMT) x Period interaction, but there were no significant interactive effects of Beach Management Type and Period (Table 3). The pairwise tests indicted that in both habitats the size compositions of clams on Sandon and Illaroo (non-fished beaches) differed between each period (Figs 3 and 4). For the two commercially fished beaches, the patterns were more complex; across both habitats the size compositions of clams on Ten Mile differed between early and late harvest, whereas on Smokey the before period differed to early and late harvest (which did not differ).

**Table 3:** Results of multivariate PERMANOVAs comparing the size compositions of clams across commercially fished and non-fished beaches before, early and late harvesting.

A. Dry Habitat	df	MS	Pseudo-F	P(perm)	Unique Perms	P(MC)	CoV%
Beach Management Type	1, 2	1866.9	0.442	1.000	3	0.641	0.0
Period	2, 4	6190.7	2.828	**0.061**	999	0.113	12.5
Beach(BMT)	2, 4	4222.8	4.854	**0.004**	998	**0.007**	11.7
BMT x Period	2, 60	4733.6	2.162	0.132	999	0.163	0.1
Beach(BMT) x Period	4, 60	2189.4	2.517	**0.012**	997	**0.026**	12.4
Residual	60	869.9					63.3
Total	71						
B. Swash Habitat	df	MS	Pseudo-F	P(perm)	Unique Perms	P(MC)	CoV%
Beach Management Type	1, 2	11434.0	0.728	0.649	3	0.544	0.0
Period	2, 4	13542.0	3.195	**0.018**	999	**0.038**	17.3
Beach(BMT)	2, 4	15702.0	10.653	**0.001**	999	**0.001**	22.1
BMT x Period	2, 52	3573.0	0.843	0.597	997	0.556	0.0
Beach(BMT) x Period	4, 52	4239.0	2.876	**0.002**	998	**0.004**	7.4
Residual	52	1473.9					53.2
Total	63						

df = degrees of freedom, MS = mean square, Pseudo-F = F-ratio, P(perm) = permutation based P-value, Unique perms = number of unique permutations, P(MC) = Monte Carlo simulation P-value, CoV% = component of variation percentage

The Beach Management Type x Period and Beach(BMT) x Period interaction terms if significant identify possible effects of fishing and management zoning

Across all beaches two sizes classes of clams (10–25 and 40–60 mm SL) were prevalent in samples taken in the swash, whereas the smaller size class was generally less prevalent in the dry (Figs 3 and 4). In general terms, greater proportions of small juveniles (< 25 mm SL) were present across all beaches, particularly in the swash habitat, in the early and late harvesting periods than before harvesting (Figs 3 and 4). The notable exception to this was the dry habitat on Smoky and Illaroo. The length composition of clams on Smoky was truncated at 55 mm across both habitats and all three periods.

## Discussion

Populations of clams on all four beaches were inherently variable across both habitats with significant differences in densities consistently occurring across individual sites sampled each day, as well as among days sampled within each period on each beach. Moreover, the components of variation were consistently greatest across the smallest spatial scale sampled; among replicate samples taken at each site on each day and they were also generally high for the factors Site and Day. These results exemplify the need for future assessments of beach clams to adequately account for small-scale variability in sampling strategies to avoid potential confounding of larger scale comparisons [33]. Small-scale spatial and temporal variability is not uncommon in benthic assemblages [41–43] and was expected; previous sampling over a hierarchy of scales identified that variability in the densities of clams was consistently greatest across the smallest spatial and temporal scales examined [33]. The ecological processes driving such small-scale variability require determination using appropriate sampling strategies and experimentation.

Despite the prevalence of small-scale variability, some significant differences in the densities and sizes of clams among individual beaches were evident, but there were no overall differences detected between the two commercially fished versus the two non-fished beaches from before to during the harvesting period. This result contrasted expectations and that often observed between protected versus non- and partially-protected areas in other systems [18,44,45]. Fishing closure effects on organisms, including beach clams, can be rapid and manifest within 1–3 years [24,46], with this study commencing 1-year post management implementation. Nevertheless, there were no detectable reductions in densities and truncation of size compositions of clams on fished compared to non-fished beaches throughout harvesting even though during the study approximately 4,300 and 17,800 kg of clams were reportedly harvested from Ten Mile and Smoky beaches, respectively. These levels of commercial harvests combined with the daily trip limit of 40 kg per-fisher may have limited the potential manifestations of fishing on populations, highlighting the difficulties in determining the potential effects of current fishing levels and management strategies on *D*. *deltoides*. Despite this, alternate harvest strategies could potentially be tested by allowing different daily and total catch quotas of clams across different beaches as part of a controlled management experiment. Importantly, further experimental evaluation is required to test the general applicability of closed areas and times, and their potential rotation [24], for managing the sustainable harvesting of beach clam resources elsewhere.

The overall lack of fishing-related effects in the current study was unlikely due to any potential confounding of recreational and indigenous harvesting of clams across open and closed beaches. Very few non-commercial fishers were observed collecting clams during sampling and the current levels of harvesting from these two sectors is considered to be low. Nevertheless, quantification of levels of clam harvests from these sectors and variability among beaches is required for a greater understanding of more global effects of human exploitation on clams.

It was further unlikely that the study beaches were not representative of commercially fished and non-fished beaches, or that statistical power issues prevented detection of fishing impacts. Twelve beaches were reported commercially fished throughout NSW in 2013, with total harvests ranging from 200 to 17,800 kg across individual beaches. Eight beaches had reported total harvests exceeding 2,000 kg. Clams were typically harvested each permitted month and across the swash and dry habitats on both fished study beaches. Although statistical detection of beach management type effects may have been limiting (see methods), the data indicated that variability in densities and sizes of clams among individual beaches was greater than that observed at the higher grouping of beach management type.

The commercial harvesting of clams does not occur evenly along and across beaches, as it is dependent on clam aggregations, ease of access and avoidance of conflict with other beach users (personal observations) [24]. Thus, any potential effects of harvesting on the densities and sizes of clams may be manifest only across small spatial scales in the immediate vicinity of actual harvesting (i.e. digging). Moreover, areas of fishing intensity on beaches often vary across habitats and with time and any potential impacts of harvesting may persist for only a small temporal period (e.g. single tidal phase). The active and passive movements of clams along and across [47–50], and potentially between beaches may further mitigate, or confound detection of, any effects of fishing on populations at the level of beaches or zones within beaches [33]. Knowledge of such movements and relationships with ecological processes could help clarify potential harvesting-related impacts on clams.

There were no detectable or observable effects of commercial harvesting on the size composition of clams across beaches from before to early or late harvesting season. The only notable difference among beaches was the absence of large clams > 55 mm SL across both habitats on Smoky, but this was evident before, early and late harvesting. Whilst this particular feature could be an artifact of historic fishing activities, it could also be due to differing growth and mortality schedules of clams on Smoky compared to the other beaches studied. Spatially explicit differential growth rates and concomitant size compositions of other clams and benthic molluscs, such as abalone and mussels, are common [51–55]. An understanding of the spatio-temporal levels of plasticity in the growth and longevity of clams and their potential relationships with biotic and abiotic processes of the beach environment could assist in determining potential impacts of fishing on populations as well as the resilience and responses of clams to differing levels of harvesting.

The recruitment of small clams (< 25 mm SL) was evident across both habitats on the fished and non-fished beaches during early and late harvesting. This timing concurs with the predominant austral winter/spring spawning of the species [26]. Small clams were most prevalent in the swash, suggesting they are mostly distributed across the lower zones of beaches, as reported for other clam species [47,56]. This could potentially be a mechanism to reduce predation and resource competition [57]. Potential effects of harvesting activities on small clams could potentially differ between habitats, and this remains an important avenue of research.

The overall lack of differences in the densities and sizes of clams between the commercially fished and non-fished beaches from before to during harvesting probably resulted from clams responding to a suite of ecological processes and natural perturbations operating independently on each individual beach. For example, abiotic factors such as beach profiles, wave conditions and storm events [49,58], in combination with biotic processes such as levels of predation, quantity and quality of food resources and competitive interactions among fauna [31,57,59,60], could all affect the dynamics of clam populations on each individual beach in different ways. Such environmental variability may have confounded comparisons across management units. Unfortunately, the ecology of *D*. *deltoides* has been little studied to help unravel such complexities and potential relationships with harvesting. Sampling clams across beaches with different management arrangements over several years may be necessary to ascertain the potential effects of fishing on populations as opposed to natural environmental processes [61,62].

Although there were no identifiable effects on clams of commercial harvesting here, harvesting could be having more widespread effects across the entire stock, as reported for other exploited species [23,24]. Indeed, this could have particularly been the case with the previous unlimited harvesting of clams by all sectors across many beaches, which may have impacted total reproductive output and concomitant levels of recruitment and population replenishment across non-fished as well as fished beaches. A strong genetic connectivity exists among clam populations along eastern Australia, suggesting high exchange of larvae among beaches [63]. However, stock-recruitment relationships and the source-sink dynamics of larvae are unknown, and are thus potential areas of future research. The current management restrictions on clam harvesting may potentially allow populations to rebuild, but this could take some time to manifest. Unfortunately, long-term closures to clam harvesting elsewhere have in some cases had minimal impacts on restoring populations due to other environmental perturbations, namely climate variability and associated effects on recruitment pulses and large-scale mortalities [23,24,62].

Future field-based studies that test alternate management arrangements may help refine and determine the most suitable harvesting strategies for this particular species and assist in developing appropriate strategies for the sustainable harvesting of beach clams elsewhere. The potential impacts of commercial harvesting of clams on other organisms and the broader beach ecosystem have not been examined, but they need to be considered for the holistic management of sandy beaches.
